# Recyclable nanoscale zero-valent iron-based magnetic polydopamine coated nanomaterials for the adsorption and removal of phenanthrene and anthracene

**DOI:** 10.1080/14686996.2016.1246941

**Published:** 2017-01-06

**Authors:** Jing Li, Qingxiang Zhou, Yongli Liu, Man Lei

**Affiliations:** ^a^ Beijing Key Laboratory of Oil and Gas Pollution Control, China University of Petroleum Beijing, Beijing, P.R. China

**Keywords:** Magnetic polydopamine nanoparticles, adsorption, phenanthrene, anthracene, magnetic separation

## Abstract

In this study, nanoscale zero-valent iron nanoparticles (NZVIs) were coated with silica and polydopamine using a two-step process. The coated nanoparticles were applied as adsorbents for removal of two common polycyclic aromatic hydrocarbons pollutants, phenanthrene (PHE) and anthracene (ANT) from aqueous system. Adsorption kinetics followed a pseudo-second-order model. Isotherms and thermodynamics were investigated and the results indicated that the adsorption process fit best to the Freundlich model and exhibited the characteristics of an exothermal physical adsorption process. Owing to their superparamagnetic characteristics and stability, these adsorbents could be easily collected and recycled for reuse.

## Graphical abstract

**Figure:**
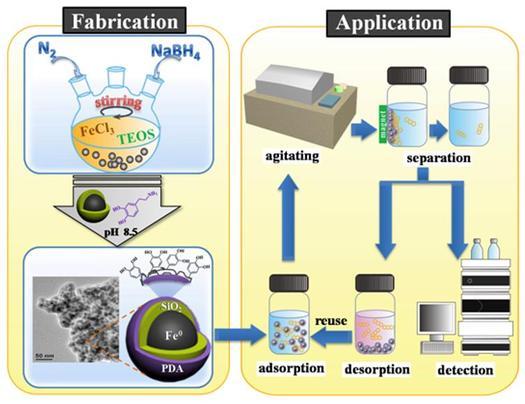

## 1. Introduction

Polycyclic aromatic hydrocarbons (PAHs) constitute a category of typical environmentally-persistent pollutants.[1] They are of particular interest to researchers due to their teratogenic, carcinogenic, and mutagenic potential.[2] While PAHs originate from both natural and anthropogenic processes, the incomplete combustion of hydrocarbon fuel currently contributes the highest proportion of environmental PAHs.[3] Furthermore, water contaminated by PAHs poses significant potential risks to human and ecological health.[4]

Remediation methodologies for a variety of pollutants generally include physical removal, chemical transformation, and biological degradation.[5] However, chemical transformation of PAHs tends to produce harmful byproducts, while biological degradation of PAHs is ineffective due to their stability.[6] Fortunately, owing to their hydrophobic properties, adsorption of PAHs with organic matter or other adsorbents has been conducted to effectively remove PAHs from aquatic systems.[7–9] Subsequently, several research groups have applied carbon materials, including graphene and carbon nanotubes, to PAHs adsorption.[10–13] However, fabrication of carbon materials is complex and these materials are relatively harmful to the human body.[14] Since the convenient separation and recovery of adsorbents has been a longstanding challenge in adsorption applications, scientists have begun to introduce magnetism into adsorbent-based systems. The popular superparamagnetic materials can be easily separated from an aqueous solution by applying an external magnetic field which makes the recycle of adsorbents possible.[15] Using this methodology, Huang and Keller [16] successfully removed methyl orange with a surfactant-functionalized magnetic nanomaterial.^[16]^ Yan et al. [17] prepared magnetic biochar/ZnS composites and demonstrated them to be promising adsorbents for Pb(II) removal^[17]^. Wang et al. [18] used Fe_3_O_4_ nanoparticles encapsulated in a SiO_2_-graphene composite to enrich and isolate trace PAHs from water for HPLC analysis,^[18]^ and Torabian et al. [4] used 3-mercaptopropyltrimethoxysiline and allyl glycidyl ether-grafted Fe_3_O_4_ nanoparticles for PAHs removal^[4]^. It is apparent that the combination of magnetic nanoparticles and other functionalized nanostructures shows great promise for effective removal of pollutants.

Nanoscale zero-valent iron particle (NZVI)-based materials are an example of superparamagnetic materials. They have been used instead for the degradation of organic or inorganic pollutants.[19–21] Limitations aside, NZVIs still possess great potential for use in magnetic separation, as it has been reported that saturation magnetization values could potentially exceed 200 emu g^–1^ for α-Fe.[22,23] In order to make full use of NZVIs, bare NZVIs require modifications to increase their stability and selectivity for ultimate improvement of their adsorption ability. In this vein, Guo et al. [24] and Motamedi et al. [25] prepared composites of Fe nanoparticles encapsulated in reduced graphene oxide (Fe@r-GO) for contaminant removal and achieved some success^[24], [25]^. Unfortunately, their adsorbent’s fabrication procedure, especially regarding GO synthesis, was exceedingly complex and expensive. Another promising material, polydopamine (PDA), is a type of multifunctional biomimetic material. PDA’s numerous functional groups (e.g. catechol) can facilitate adsorption of virtually all kinds of substrates, including metals, ceramics and synthetic polymers.[26–29] PDA has also been shown to be a practical material for use in coating magnetic cores to improve their adsorption of organic pollutants, including PAHs, from water.[30,31] While the mechanism of adsorption is still unknown, π–π stacking interactions appear to contribute to adsorption.[30] From prior studies regarding determination of trace pollutants, the synergistic combination of magnetic nanoparticles and PDA modification is a strategy that holds promise for use in adsorption and removal of pollutants.

To date, few research groups have studied the synthesis of nanoscale zero-valent iron (NZVI)-based magnetic hybrid materials for use as adsorbents or tested their suitability as adsorbents for removal of PAHs from water. In the present work, we used a simple two-step method to prepare a magnetic core-shell material (Fe@SiO_2_@PDA) with NZVI as a magnetic core, followed by coating of the NZVI particles with a double shell of silica as the inside layer and polydopamine as the outside layer. The iron core was fabricated by borohydride reduction [32] then coated with SiO_2_ via the hydrolysis and condensation of tetraethylorthosilicate (TEOS) onto the outer wall of the NZVI nanoparticles. PDA was then applied to the core to form an outer coating via the self-polymerization of dopamine.[33]

In the work described here, the adsorption mechanism of two PAHs to Fe@SiO_2_@PDA nanoparticles in water was elucidated. The PAHs chosen for study are two members of the 16 priority pollutants PAHs by the US Environmental Protection Agency.[34] Phenanthrene (PHE) and anthracene (ANT), each with three benzene rings, were chosen as target compounds for testing of their adsorption to modified NZVI nanoparticles and their properties and molecular structure are listed in Table 1. Factors affecting adsorption efficiency were investigated, including solution pH, ionic strength, concentration of humic acid (HA), and the initial concentration of target compounds. Next, we conducted adsorption kinetics studies under optimal conditions, followed by isotherm and thermodynamics studies. The adsorption kinetics and isotherm results supported a pseudo-second-order model and Freundlich model, respectively. Thermodynamic study verified the adsorption to be a spontaneous exothermal physical adsorption process. In addition, these adsorbents exhibited sufficient stability and retained their adsorptive ability upon regeneration.

**Table 1.** Selected properties of phenanthrene and anthracene.

Compound	Abbreviation	Structure	MW* (g/mol)	π electron	log K_ow_*	C_s_*(mg/g)	References
Phenanthrene	PHE		178.2	14	4.57	1.29	[7, 13, 35, 36]
Anthracene	ANT		178.2	14	4.54	0.073	[37]

* MW: molecular weight; K_ow_: octanol–water partition coefficient; C_s_: water solubility.

## 2. Experimental

### 2.1. Reagents

Standards of PHE (95%), ANT (99%), tetraethyl orthosilicate (TEOS, 98%) and polyvinylpyrrolidone (PVP) were purchased from Aladdin Industrial Co. Ltd (Shanghai, China). Dopamine hydrochloride (99%) was purchased from J&K Scientific Co. Ltd (Beijing, China). HPLC grade acetonitrile and methanol were obtained from Oceanpak Alexative Chemical Ltd (Gothenburg, Sweden). Ferric chloride hexahydrate (FeCl_3_·6H_2_O, AR), sodium borohydride (NaBH_4_, AR), and humic acid (HA, CP) were purchased from Guangfu Chemical Reagent Company (Tianjin, China). Sodium hydroxide (NaOH), hydrochloric acid (HCl, 37%) and sodium chloride (NaCl) were purchased from Beijing Chemical Reagent Company (Beijing, China); they were all of analytical grade and used without further purification. Standard stock solutions (100 mg l^−1^) of three PAH compounds were prepared in methanol prior to use. Working solutions were prepared by mixing an appropriate amount of the stock solution with ultrapure water. The concentration of organic solvent (methanol) in the final solutions was kept below 0.2% (v/v). All the standard solutions were stored at 4°C and protected from light. Ultrapure water was used during our experiments.

### 2.2. Preparation of Fe@SiO_2_@PDA

**Figure 1.**
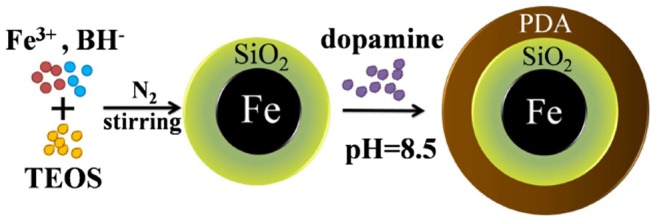
Schematic illustration of the synthesis of Fe@SiO_2_@PDA nanocomposites.

As the first step in preparation of Fe@SiO_2_@PDA, silica-coated NZVI particles were synthesized using a one-step method, as depicted in Figure 1 [38] and similar to our previous study.[39] Briefly, particles were synthesized through a modified process of reduction of ferric ion (Fe^3+^) by borohydride, along with the formation of silica. The nanoscale zero-valent iron was formed according to the following reaction:

$$4{\text{Fe}}^{3+}+3{\text{BH}}_{4}^{-}+9{\text{H}}_{2}\text{O}\to 4{\text{Fe}}^{0}↓+3{\text{H}}_{2}{\text{BO}}_{3}^{-}+12{\text{H}}^{+}+6{\text{H}}_{2}↑$$

At the same time, a silica shell was formed through hydrolysis and condensation of TEOS. In this reaction, NaBH_4_ acted as a substitute for the traditional ammonia catalyst, because the pH of the reactant increased with the addition of NaBH_4_ to alkaline pH. After 2 h, particulate materials were collected using a permanent magnet and rinsed several times with distilled water and absolute ethanol. The collected samples were vacuum-dried at 50°C overnight and ground.

The PDA coating was achieved using a straightforward dopamine self-polymerization process, which could be safely performed in most laboratories using readily available chemical reagents and equipment. Prepared Fe@SiO_2_ (200 mg) and 50 mg dopamine hydrochloride were added to 100 ml 10 mM Tris-HCl buffer at pH 8.5. Next, the mixture solution was mechanically, but gently, stirred for 16 h at room temperature. The final products were washed with distilled water and vacuum-dried at 50°C overnight, creating the modified NZVI particles that we denote as Fe@SiO_2_@PDA. It has been reported that PDA bonded to silica because the hydroxyl or carbonyl groups in polydopamine could form a hydrogen bond network with the oxygen atoms on the surface of Fe@SiO_2_.[27]

### 2.3. Characterization of Fe@SiO_2_ and Fe@SiO_2_@PDA

A mechanical agitator (Kexi J-J1, Jintan, China) and vacuum drying oven (Kewei, Beijing, China) were used to prepare the adsorbent materials. Water-bathing constant temperature vibrator (DSHZ 300A, Taicang, China) was used for adsorption experiment; Agilent 1260 HPLC (Santa Clara, CA, USA) was used for concentration analysis; transmission electron microscopy (TEM, JEM-2100 LaB_6_, Akishima-shi, Japan) was used to observe morphology. Energy dispersive X-ray spectroscopy (EDS, Trident XM4, Mahwah, NJ, USA), X-ray diffraction (XRD, Rigaku D/max-2500, Tokyo, Japan) and X-ray photoelectron spectroscopy (XPS, Thermo Fisher K-Alpha, New York, NY, USA), Fourier transform infrared spectroscopy (FTIR; 8400S, Shimadzu Co. Ltd., Kyoto, Japan) were used to analyze the composition. The Brunauer-Emmett-Teller (BET) surface areas were measured by N_2_ adsorption and desorption (Quantachrome Instruments, Boynton Beach, FL, USA). Vibrating sample magnetometer (VSM, Lake Shore 7410, Lake Shore Cryotronics, Inc., Westerville, OH, USA) was used for magnetic measurement.

TEM analysis was performed at an operating voltage of 200 kV. The prepared particles were dispersed in ethanol by ultrasonication and dripped onto a carbon foil supported on a duplex copper grid. EDS analysis was conducted along with TEM observation. XRD measurements were performed with Cu Kα radiation (λ = 1.5418 Å) at 40 kV and 200 mA. The scan speed was 4° min^–1^ and in the range of 10–90°. XPS measurements provided the elemental (Fe, Si, C, N, O) contents and valence states of sample surfaces up to a depth of about 3–5 nm. The binding energies of the photoelectrons were calibrated by the aliphatic adventitious hydrocarbon C(1s) peak at 284.6 eV. FTIR (8400S) was also applied for a further identification of functional groups in the coating using KBr pellets in the wave numbers ranging from 4000 to 500 cm^−1^. Magnetic measurements (hysteresis loop) were performed with a VSM (Lake Shore 7410) in the magnetic field range of –20,000 to 20,000 Oe.

### 2.4. Adsorption experiment

In this work, The Fe@SiO_2_@PDA nanoparticles were used as adsorbents in an adsorption experiment for removal of phenanthrene (PHE) and anthracene (ANT). Experiments were carried out in 20 ml polytetrafluoroethylene (PTFE) screw cap brown vials sealed with aluminum foil in a thermostatic bath with constant agitation (200 rpm) at a given temperature. The background solution in the samples contained 0.01 M NaCl (~0.058%, w/v) in distilled water to maintain a constant ionic strength.[36] Either 40 mg or 20 mg of Fe@SiO_2_@PDA was dispersed in 20 ml of 0.5 mg l^−1^ PHE or 0.3 mg l^−1^ ANT solutions, respectively, in 20 ml brown vials. The volume of organic solvent in the background solution was controlled to be less than 0.2% (v/v) to avoid the co-solvent effect.[13] The pH, ionic strength, humic acid concentration and initial concentration for PHE and ANT) were varied to study their respective influences on the adsorption ability of the nanoparticles for PHE and ANT.

The amount of PAHs adsorbed by the sorbent at equilibrium (q_e_) and adsorption efficiency (%) were calculated according to the following equations (Equations (1) and (2)):

(1) $${\text{q}}_{\text{e}}=\frac{\text{V}\times ({\text{C}}_{0}-{\text{C}}_{\text{e}})}{\text{M}}$$

(2) $$\text{Adsorption efficiency}=\frac{{\text{C}}_{0}-{\text{C}}_{\text{e}}}{{\text{C}}_{0}}\times 100\%$$

where C_0_ and C_e_ are the initial and equilibrium concentrations of target PAHs in the reaction solution (mg l^−1^), respectively. V is the volume of solution (L); M is the mass of sorbent (g). q_e_ is the equilibrium concentration of contaminants adsorbed on the unit mass of Fe@SiO_2_@PDA (mg g^−1^).

An Agilent 1260 HPLC system with a fluorescence detector (FLD) was used for concentration analysis. A Shim-pack VP-ODS column (4.6 × 250 mm, 4.6 μm) was used for separation. The mobile phase was a mixture of methanol and water (90:10, v/v), the flow rate and the column temperature were controlled at 1 ml min^−1^ and 30 °C, respectively. The excitation and emission wavelengths were set as 250 nm and 364 nm for PHE, and 250 nm and 376 nm for ANT, respectively.

### 2.5. Adsorption kinetics experiment

Adsorption kinetics is an item which reflects mass transfer rate in adsorption process. In this study, it was investigated with a series of independent experiments with either 40 mg (for 0.5 mg l^−1^ PHE) or 20 mg (for 0.4 mg l^−1^ ANT) adsorbents at 298 K. The time intervals in adsorption process were kept from 5 min to 12 h in the optimizing step. The pseudo-first-order model (Equation (3)), pseudo-second-order model (Equation (4)) and intraparticle diffusion model (Equation (5)) were used to fit the experiment data.[40,41]

(3) $$\ln\left({\text{q}}_{\text{e}}-{\text{q}}_{\text{t}}\right)={\text{lnq}}_{\text{e}}-{\text{k}}_{1}\text{t}$$

(4) $$\frac{\text{t}}{{\text{q}}_{\text{t}}}=\frac{1}{{\text{k}}_{2}{\text{q}}_{\text{e}}^{2}}+\frac{1}{{\text{q}}_{\text{e}}}\text{t}$$

(5) $${\text{q}}_{\text{t}}={\text{k}}_{3}{\text{t}}^{0.5}+\text{C}$$

where k_1_ (min^−1^), k_2_ (g mg^−1^ min^−1^) and k_3_ (mg g^−1^ min^0.5^) are the pseudo-first-order, pseudo-second-order and intraparticle diffusion rate constants, while q_e_ and q_t_ (mg g^−1^) are the adsorption capacity at equilibrium and after time t (min) respectively. The value of k_1_, k_2_ and k_3_ were obtained from the slope of the plot of ln(q_e_ – q_t_) against t, t/q_t_ against t, and q_t_ against t^0.5^.

### 2.6. Adsorption isotherms experiment

To investigate the adsorption isotherms, solutions with various initial concentrations were treated with the same procedure as above at three different temperatures (298, 303 and 308 K). The equilibration time was set at 10 h uniformly, which was sufficient to reach the adsorption equilibrium. Adsorption isotherm data were investigated with the Langmuir (Equation (6)), Freundlich (Equation (7)), Temkin (Equation (8)) and Dubinin–Radushkevich (D–R; Equation (10)) models.[6,42,43] The Langmuir model assumes that the adsorbent surface is homogeneous and all the binding sites are equal without any interaction between adsorbed substances.[6] Conversely, the Freundlich model represents a heterogeneous adsorption with a non-uniform distribution of adsorptive sites. The Temkin model also represents a heterogeneous adsorption process, and adsorbates in this system have interaction with each other. The D–R equation is a semi-empirical equation, generally used to analyze the adsorption energy of the adsorption process, and to see the adsorption mechanism by calculating the mean free energy.[39,42,44] These models can be expressed by the serials of equations below:

Langmuir model

(6) $$\frac{{\text{C}}_{\text{e}}}{{\text{q}}_{\text{e}}}=\frac{{\text{C}}_{\text{e}}}{{\text{q}}_{\text{m}}}+\frac{1}{{\text{K}}_{\text{L}}{\text{q}}_{\text{m}}}$$

Freundlich model:

(7) $${\text{lnq}}_{\text{e}}=\frac{1}{\text{n}}{\text{lnC}}_{\text{e}}+{\text{lnK}}_{\text{F}}$$

Temkin model:

(8) $${\text{q}}_{\text{e}}=\text{BlnA}+{\text{BlnC}}_{\text{e}}$$

(9) B=RT/b

D–R model:

(10) $${\text{lnq}}_{\text{e}}={\text{lnq}}_{\text{m}}^{\prime }-{\text{K}}_{\text{DR}}\varepsilon^{2}$$

(11) $$\varepsilon =\text{RTln}\left(1+\frac{1}{{\text{C}}_{\text{e}}}\right)$$

(12) $${\text{E}}_{\text{DR}}={\left(2{\text{K}}_{\text{DR}}\right)}^{-1/2}$$

where C_e_ is the concentration at equilibrium (mg l^−1^), q_m_ is the maximum adsorption capacity (mg g^−1^) and q_e_ is the adsorption amount at equilibrium (mg g^−1^). K_L_ (l mg^−1^) is the Langmuir constant, can be determined from a linear plot of C_e_/q_e_ versus C_e_; K_F_ (l g^−1^) is the Freundlich constant and n is a dimensionless factor of heterogeneity, which can be determined from a linear plot of ln q_e_ versus lnC_e_; A (l g^−1^) and b (J mol^−1^) are the equilibrium binding constants and Temkin constants, respectively, and can be determined from a linear plot of q_e_ versus lnC_e_; q_m_′ is the D–R adsorption capacity (mol kg^−1^), K_DR_ is the D–R constant related to the free energy(mol^2^ kJ^−2^) and ε is the Polanyi potential. q_m_′ and K_DR_ can be calculated from the intercepts and slopes of the plots of ln(q_e_) versus ε^2^ and E_DR_ (kJ mol^−1^) is the mean free energy of adsorption. In addition, R (8.314 J mol^−1^ K^−1^) is the universal gas constant and T (K) is the absolute solution temperature.

### 2.7. Thermodynamic study

The thermodynamic parameters Gibbs free energy (ΔG^0^), enthalpy change (ΔH^0^), and entropy change (ΔS^0^) are useful in analyzing the sorption mechanism.[45] They were calculated at temperatures of 298, 303 and 308 K respectively by the following equations:

(13) $${\text{K}}_{0}=\frac{{\text{a}}_{\text{s}}}{{\text{a}}_{\text{e}}}=\frac{{\text{v}}_{\text{s}}}{{\text{v}}_{\text{e}}}\frac{{\text{C}}_{\text{s}}}{{\text{C}}_{\text{e}}}$$

(14) $$\Delta {\text{G}}^{0}=-{\text{RT\textbackslash{},ln\textbackslash{},K}}_{0}$$

(15) $${\text{lnK}}_{0}=\frac{\Delta {\text{S}}^{0}}{\text{R}}-\frac{\Delta {\text{H}}^{0}}{\text{RT}}$$

(16) $$\Delta {\text{G}}^{0}=\Delta {\text{H}}^{0}-\text{T}\Delta {\text{S}}^{0}$$

In these equations, R is the gas constant (8.314 J mol^−1^ K^−1^), and T is the absolute temperature in Kelvin. Based on the approximate equation of Equation (13), lnK_0_ can be calculated from the slope and intercept of ln(C_e_/q_e_) versus q_e_ plots.[46,47] ΔH^0^ and ΔS^0^ were obtained from the slope (ΔH^0^/R) and intercept (ΔS^0^/R) of the ln K_0_ versus 1/T plot. ΔG^0^ is the fundamental criterion to estimate if a reaction is spontaneous at a given temperature from its value. A negative value shows a spontaneous process and the positive one presents an opposite result.[48]

## 3. Results and discussion

### 3.1. Physicochemical characterization

**Figure 2.**
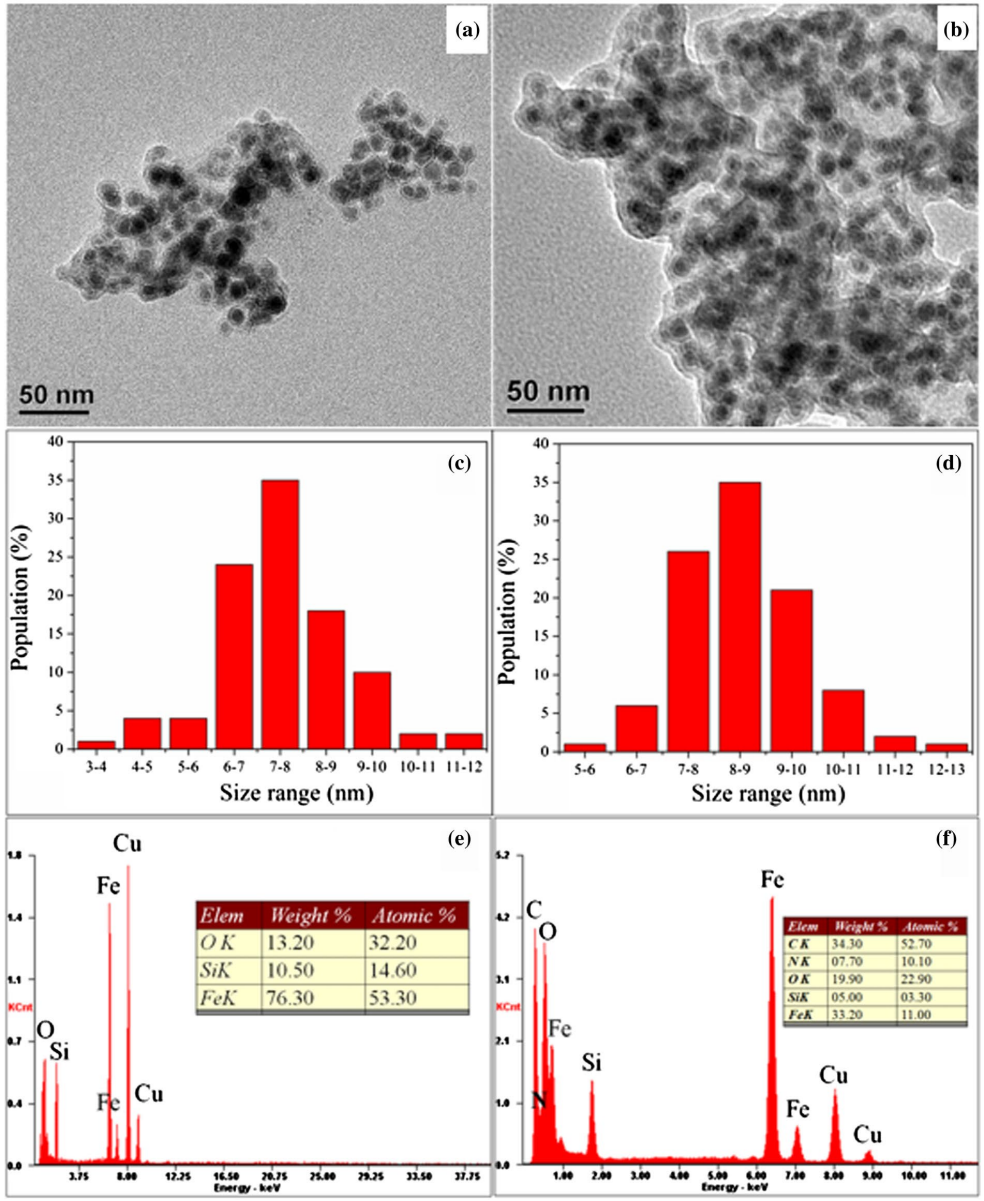
TEM images of (a) Fe@SiO_2_, (b) Fe@SiO_2_@PDA; size distribution histograms of (c) Fe@SiO_2_ and (d) Fe@SiO_2_@PDA; and corresponding EDS spectra of (e) Fe@SiO_2_ and (f) Fe@SiO_2_@PDA.

The TEM images of Fe@SiO_2_ and Fe@SiO_2_@PDA are presented in Figure 2(a) and (b), respectively. Fe@SiO_2_ exhibits thin layers of SiO_2_(~2–3 nm thickness) with distinguishable dark portions inside. Fe@SiO_2_@PDA exhibits an additional layer outside (~3–6 nm thickness). Unlike most NZVI particles reported, our silica-coated NZVIs present no chainlike aggregated structures or obvious oxidation phenomena, due to the PDA coating of each silica shell.[38,49] The size distribution histograms (Figure 2(c) and (d)) show that all the NZVI core sizes are below 13 nm, 80% of which are in the range of 6–11 nm. Iron-based nanoparticles of these dimensions are supposed to be superparamagnetic, since their sizes are below the critical size.[22,23,50] The elemental composition of products was analyzed using EDS. In Figure 2(e) and (f), the elements Fe, Si, O, C and N in these samples and their proportions are listed. Fe@SiO_2_ comprised Fe, Si, O, and other trace elements, while Fe@SiO_2_@PDA comprised C and N, in addition to the above elements (Figure 2(f)), reflecting the presence of PDA. BET measurements were applied to analyze the potential adsorptive property of these adsorbents, since adsorption is mainly determined by particle sizes, porosity, and dispersity. Fe@SiO_2_@PDA is found to have a specific surface area of 58.9681 m^2^ g^−1^, which is higher than that of Fe@SiO_2_ (37.0618 m^2^ g^−1^). These results indicate that successful coating with PDA had been achieved, with alleviation of aggregation through steric effects. Moreover, these values were in agreement with other studies.[49,51]

**Figure 3.**
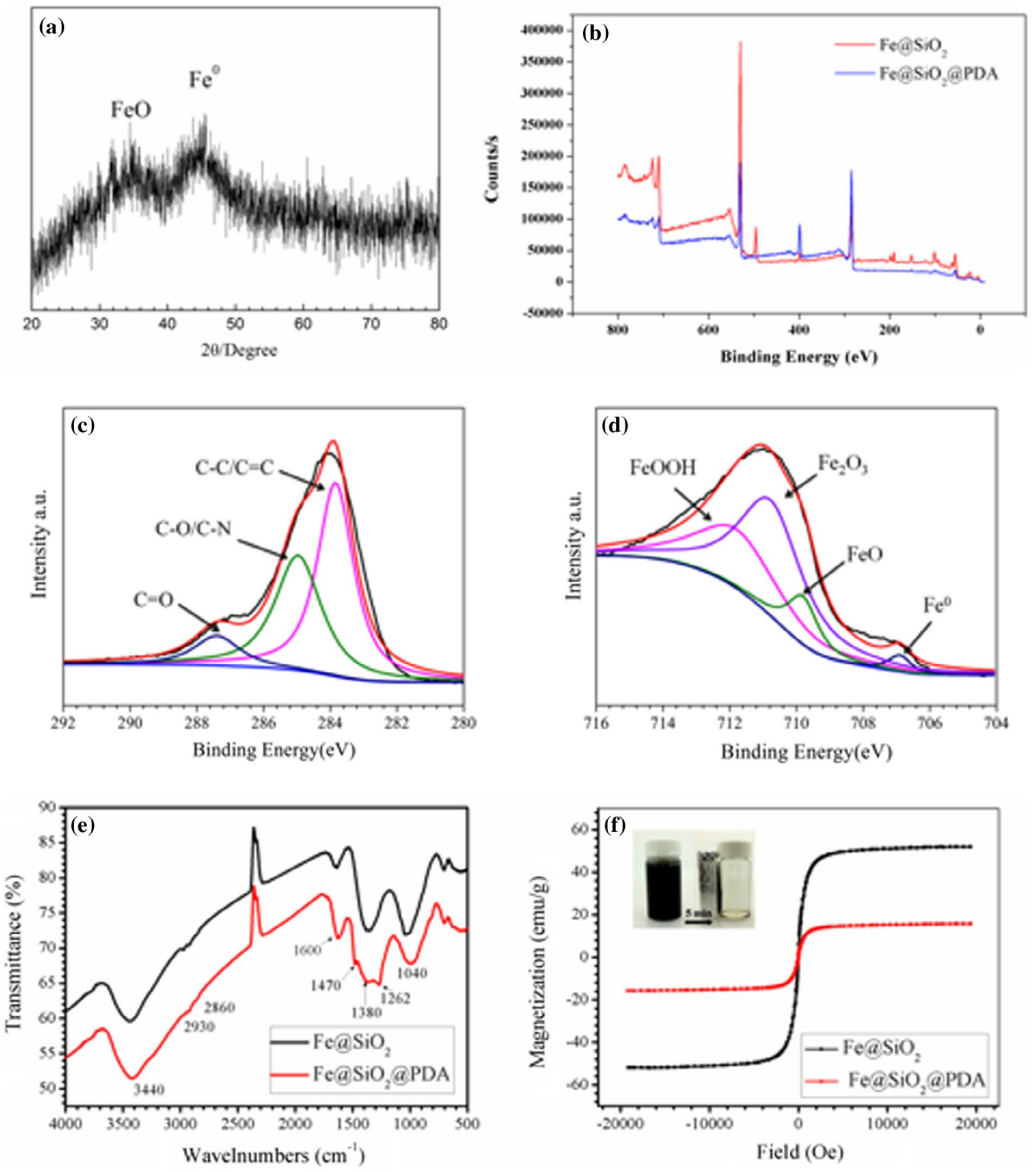
XRD pattern of Fe@SiO_2_ (a); overview (b) and high-resolution XPS spectra for Fe (c) and C (d). FTIR (e) and VSM hysteresis curves (f) of Fe@SiO_2_ and Fe@SiO_2_@PDA nanocomposites.

Figure 3(a) shows the XRD patterns of the prepared hybrid nanoparticles. Two broad diffraction peaks at 30° and 44° were observed, indicating the existence of a small amount of free iron oxide and large amounts of poorly ordered, amorphous NZVI.[32] These oxides are inevitable because the coating of the silica shell with PDA is never complete. The two modified NZVIs were also analyzed by XPS (Figure 3(b–d)). Wide-scans verified the existence of Fe, Si, and O on the Fe@SiO_2_ surface and further confirmed the addition of C and N, attributable to PDA. Because only a limited depth can be analyzed, XPS spectra can only be used to explore a surface thickness of less than 10 nm. As a result, Fe and Si are almost absent from the spectra of Fe@SiO_2_@PDA. The high-resolution spectra of Fe from the Fe@SiO_2_ sample reveal the existence of zero-valent iron (Fe^0^) as a peak at 706.9 eV. The peaks at 709.8 eV, 710.7 eV, and 711.8 eV represent the existence of iron oxides, likely in the form of FeO, Fe_2_O_3,_ and FeOOH, respectively.[32,52] The limited depth that could be observed by XPS prevents reliable analysis of the constituents within the inner cores. The slight oxidization observed on the Fe@SiO_2_@PDA surface may be attributed to a local absence of coating in the SiO_2_ shell, perhaps due to the opening of micropores in it. However, PDA is adequately coated onto the surface, as exhibited using the high-resolution spectra of C in the Fe@SiO_2_@PDA sample (Figure 3(d)), since sizeable C−O, C−N, C−C, and C=C peaks are observed. This agrees with results observed after PDA coating of particles in other reports.[21]

FTIR performed over the range of wave numbers from 4000 to 500 cm^−1^ was used to identify the existence of coating material, as shown in Figure 3(e). The main bands in the IR spectrum of Fe@SiO_2_ (Figure 3(e)) could be seen as follows: the strong band centered at 1040 cm^−1^ is attributable to the asymmetric stretching frequency of Si-O-Si and the peaks at1380 and 3440 cm^−1^correspond to the hydroxyl groups on the surface of the silica shell.[30] As for the PDA-modified sample (Figure 3(e)), new peaks at ~1262 cm^−1^ and the peak enhancement at ~1600 cm^−1^ correspond to the aromatic rings in the PDA polymer, while the sharp peak at ~1420–1470 cm^−1^ is derived from the amino group.[30,53] The adsorption peaks around 2930 cm^−1^ and 2860 cm^−1^ are ascribed to the stretching vibrations of CH_2_ and CH_3_ species, respectively. Consistent with the results of XPS, FTIR spectroscopy further revealed that PDA has been decorated onto the Fe@SiO_2_ nanoparticles.

The magnetic properties were studied at room temperature using a VSM. The saturation magnetization (M_s_) values of Fe@SiO_2_ and Fe@SiO_2_@PDA were found to be 51.98 and 15.769 emu g^−1^, respectively, which were sufficient for magnetic separation (Figure 3(f)). In comparison with several similar adsorbents listed in Table 2, the saturation magnetization in our research still showed a satisfactory result. The relative loss of magnetization value in Fe@SiO_2_@PDA indicated the presence of nonmagnetic PDA. In addition, it was further confirmed that the particles exhibited reusability, as they exhibited superparamagnetic-like behavior at room temperature with small coercivity and remanence. Collectively, all of the above data convincingly support successful formation of Fe@SiO_2_@PDA.

**Table 2.** Comparison of several iron-based nanoparticles.

Material	Diameter (nm)	M_s_ (emu g^−1^)	Reference
Fe^0^@γ-Fe_2_O_3_- FeOOH@G	1–5	19	[54]
Fe_3_O_4_@SiO_2_	80–100	11.8	[55]
Fe_3_O_4_@SiO_2_@SiO_2_-C_18_	200	7.35	[55]
Fe_3_O_4_-SH	—	5.35–11.11	[56]
Fe@SiO_2_	50	12	[57]
Fe@SiO_2_	<10	51.98	Present work

G: graphene.

### 3.2. Optimization of adsorption conditions

**Figure 4.**
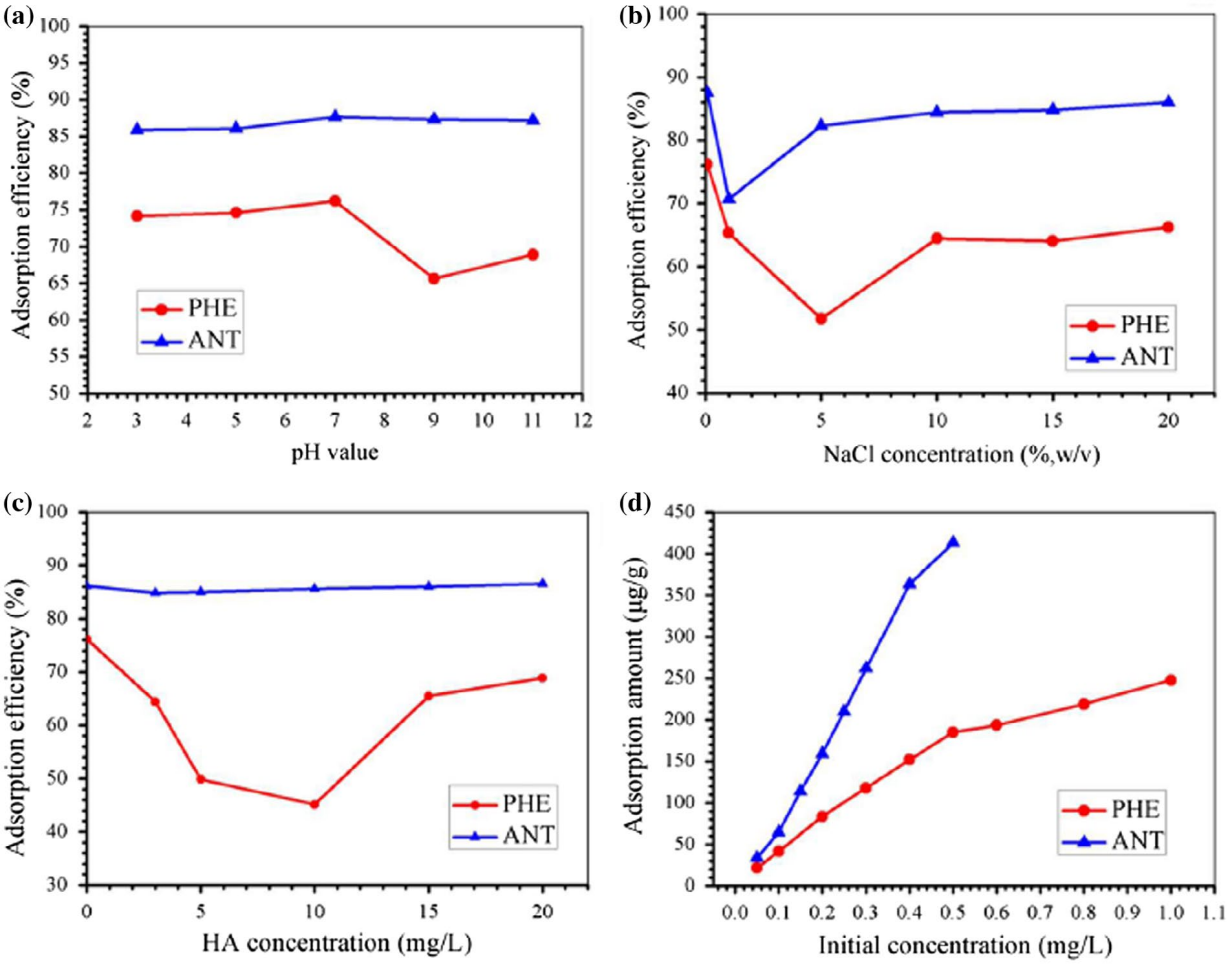
Influence of pH (a), ionic strength (b), HA concentration (c) and initial concentration (d) on the removal of PHE and ANT by the as-prepared Fe@SiO_2_@PDA.

As pH is a parameter usually considered in the adsorption process, it was investigated in the range of pH 3–11 by adjusting the sample pH with HCl and NaOH solutions. From our results (Figure 4(a)), there was almost no significant change in adsorption efficiency with change in pH for ANT because ANT is a neutral molecule unlikely to be influenced by variations in pH,[12] and the straight-line structure is easily adsorbed by benzene ring of PDA layer through π–π stacking interaction. However the adsorption efficiency of PHE slightly decreased in strong alkaline conditions because the alkali changed the surface structure of PDA layer, and the nonlinear structure of PHE had a strong steric effect which made it difficult to adsorb onto the surface of the PDA layer.

To investigate the effect of ionic strength on the adsorption efficiencies of PHE and ANT, various concentrations of NaCl were introduced into the adsorption system (via background solutions, 5, 10, 15 and 20%, w/v). The results showed that the addition of NaCl had an effect within the concentration range investigated (Figure 4(b)). A reduction in adsorption efficiency was observed between 0–5% NaCl, probably due to a salting-in effect. Adsorption efficiency increased upon further addition of NaCl until the curve leveled off just 2–12% below the 0% NaCl level of adsorption efficiency, as seen in other studies.[58] Therefore, no extra NaCl was added to the system in all subsequent experiments.

Overall humic acid (HA) reduced PHE adsorption efficiencies below the initial 76.2%, declining almost 30% as the concentration of HA increased from 0 to 10 mg l^−1^, then increasing to 68.87% in the range of 10 to 20 mg l^−1^ HA (Figure 4(c)). As for ANT, the shape of the fluctuation in the curve was similar to that of PHE, but much less pronounced, with a fluctuation amplitude less than 2%. HA inhibition of PHE removal efficiency was probably due to competition for adsorption between HA and PHE for reactive sites on the adsorbent’s surface. The increasing adsorption behavior at higher HA levels might be due to PHE adsorption by HA itself, as supported by reports of the existence of π–π interactions between HA and materials containing benzene structures.[54] With increasing HA concentration, adsorbed HA could then, in turn, provide new adsorptive sites to some extent, leading to a slight improvement in PHE removal efficiency.

Initial concentrations of analytes were also investigated to study their adsorption trends (Figure 4(d)). Due to the low solubility of ANT, its concentration was adjusted to achieve oversaturation and could decrease the solubility value after adsorption in solution. The results revealed that the adsorption amounts of PHE and ANT rose from 0.022 mg l^−1^ to 0.248 mg l^−1^ and 0.034 mg l^−1^ to 0.413 mg l^−1^, respectively, in the concentration range investigated. While an apparent decrease in adsorption amount was revealed after using specific initial PHE and ANT concentrations, these initial concentrations were considered to be turning points in adsorption capacity. This might be due to unavailability of the adsorbent’s surface.[46] Hence, 0.5 mg l^−1^ and 0.4 mg l^−1^ were kept as initial concentrations for PHE and ANT in the kinetics study, respectively.

### 3.3. Adsorption kinetics

**Figure 5.**
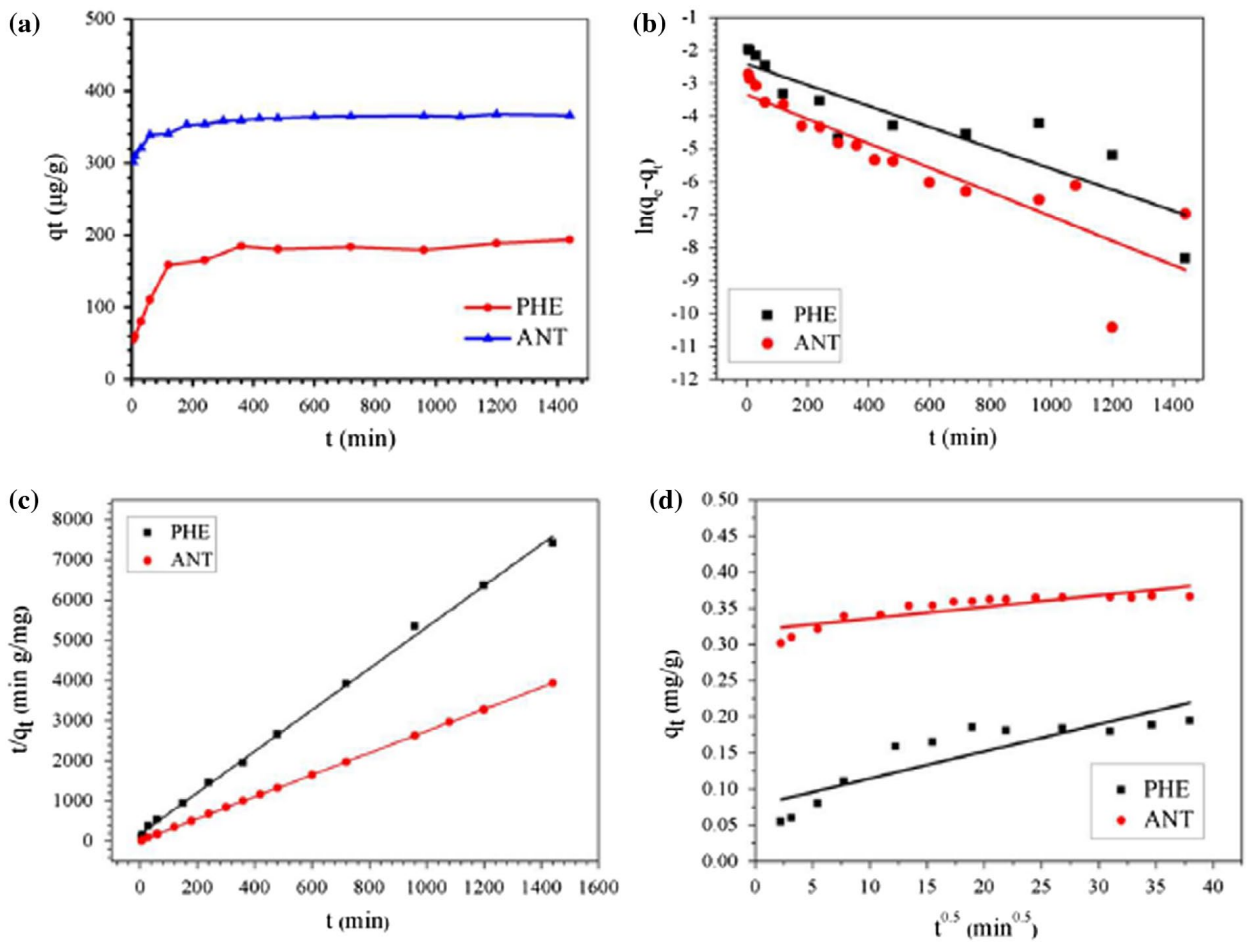
Kinetics of PHE and ANT removal by Fe@SiO_2_@PDA nanocomposites (a) and the fitted curves of pseudo-first-order model (b), pseudo-second-order model (c) and intraparticle diffusion model (d).

The adsorption behaviors of the Fe@SiO_2_@PDA towards PHE and ANT at different time intervals are depicted in Figure 5(a). From this figure, we can see that a fast adsorption process occurs during the first 2 h and gradually reaches equilibrium within 10 h. The sufficiently active sites contributed to the fast adsorption in the initial stage, then the adsorption slowed down because there were fewer active sites available. The adsorption capacities of PHE and ANT under these conditions were determined to be 0.185 and 0.367 mg g^−1^, respectively. These are higher than the adsorption capacities observed by Saad et al. [6] and Liu et al. [36]. In order to evaluate the kinetics of the adsorption process, the pseudo first-order, pseudo second-order, and intraparticle diffusion models were tested to interpret the experimental data. The values of constants and correlation coefficients (*R* ^2^) are listed in Table 3. Data suggest that the pseudo-second-order model is most appropriate, as its correlation coefficients (*R* ^2^) are the highest and the calculated adsorption amounts (q_e_) are in agreement with the actual values. Therefore, the rate-controlling step is the surface adsorption by valence forces through sharing or exchanging of electrons between adsorbent and adsorbate.

**Table 3.** Adsorption rate constants for kinetic models at 298 K pH 7.0.

Kinetic models	PHE			ANT		
	k	q_e_ (cal)	*R*^2^	k	q_e_ (cal)	*R*^2^
Pseudo-first-order	0.003 (min^−1^)	0.089(mg g^−1^)	0.795	0.003(min^−1^)	0.035(mg g^−1^)	0.772
Pseudo-second-order	0.15 (g mg^−1^ min^−1^)	0.194(mg g^−1^)	0.998	2.22(g mg^−1^ min^−1^)	0.367(mg g^−1^)	0.999
Intraparticle diffusion	0.003(mg g^−1^ min^0.5^)	–	0.767	3 × 10^−5^(mg g^−1^ min^0.5^)	–	0.740

### 3.4. Adsorption isotherms

**Figure 6.**
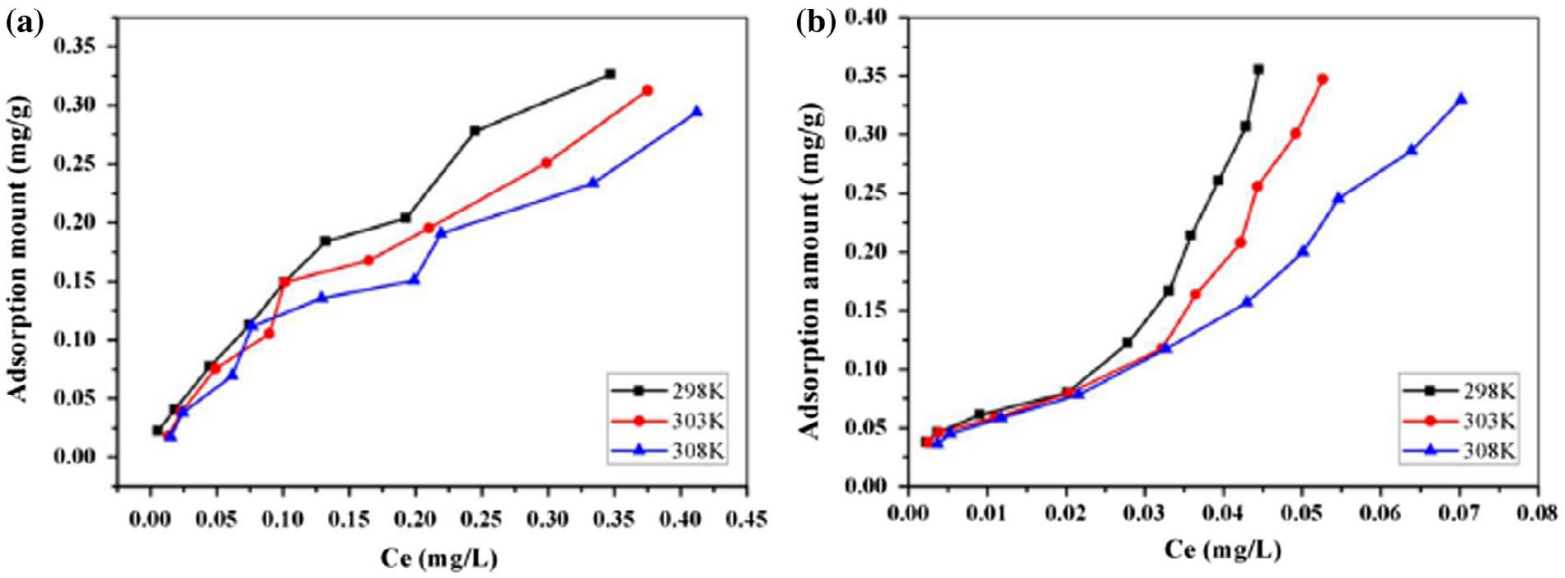
Adsorption isotherms of PHE (a), ANT (b) at 298, 303 and 308 K.

In addition to adsorption kinetics, adsorption isotherms of PHE and ANT onto composites were analyzed to explore the adsorption mechanism in greater depth. This study was performed at 298, 303, and 308 K by changing the initial concentrations within the ranges of 0.05–1 and 0.04–0.4 mg l^−1^ for PHE and ANT, respectively. Figure 6 shows the adsorption isotherm of these two selected substances. The adsorption capacity (q_e_) increased with an increase in equilibrium concentration (C_e_), while the curves could not reach a plateau even at the highest C_e_ values. This was because the C_0_ was not high enough to achieve saturation adsorption, owing to low adsorbate solubility. Four typical adsorption models, the Langmuir, Freundlich, Temkin, and D–R models, were used to simulate the adsorption isotherms of PHE and ANT onto Fe@SiO_2_@PDA. The regression parameters are presented in Table 4.

**Table 4.** Regression parameters of isotherm models.

Isotherm	Analytes	Temperature (K)	Parameters		
**Langmuir**			**K**_**L**_**(l mg**^**−1**^**)**	**q**_**m**_**(mg g**^**−1**^**)**	***R***^**2**^
	**PHE**	298	4.87	0.484	0.855
		303	2.80	0.572	0.868
		308	2.73	0.512	0.820
	ANT (low*/high*)	298	260/–14.7	0.094/–0.184	0.993/0.977
		303	216/–10.8	0.094/–0.259	0.972/0.817
		308	148/–4.71	0.101/–0.663	0.979/0.829
**Freundlich**			**K**_**F**_**(l g**^**−1**^**)**	**n**	***R***^**2**^
	**PHE**	298	0.662	1.50	0.992
		303	0.729	1.25	0.975
		308	0.614	1.28	0.965
	ANT (low*/high*)	298	0.297/118	2.99/0.528	0.996/0.978
		303	0.293/34.9	2.92/0.626	0.986/0.949
		308	0.399/7.93	2.38/0.824	0.999/0.985
**Temkin**			**A (L g**^**−1**^**)**	**b (kJ mol**^**−1**^**)**	***R***^**2**^
	**PHE**	298	120	34.9	0.847
		303	66.5	31.1	0.914
		308	61.5	33.3	0.908
	ANT (low*/high*)	298	3143/58.9	130.4/7.79	0.988/0.862
		303	2683/56.6	132.6/9.09	0.955/0.846
		308	1552/58.7	111.3/12.3	0.984/0.909
**D–R**			**q**_**m**_**′ (mol kg**^**−1**^**)**	**E**_**DR**_**(kJ mol**^**−1**^**)**	***R***^**2**^
	**PHE**	298	0.292	5	0.932
		303	0.339	4.08	0.985
		308	0.306	4.08	0.976
	ANT (low*/high*)	298	0.132/4.88	10/3.54	0.996/0.967
		303	0.128/2.54	9.13/3.54	0.974/0.929
		308	0.153/1.24	8.45/4.08	0.994/0.970

^*^ Low: C_0_ (initial concentration) < 0.1 mg l^–1^; high: C_0_ (initial concentration) ≥ 0.1 mg l^–1^.

As for PHE, the Freundlich model correlates well with the equilibrium data, indicating the adsorption processes are mainly controlled by Freundlich surface adsorption mechanisms involving heterogeneous multilayer sorption processes. The n values are greater than unity, indicating that PHE was favorably adsorbed by Fe@SiO_2_@PDA. Moreover, from the E value in the D–R model (<8 kJ mol^−1^), the adsorption is deduced to be a physical process.

**Figure 7.**
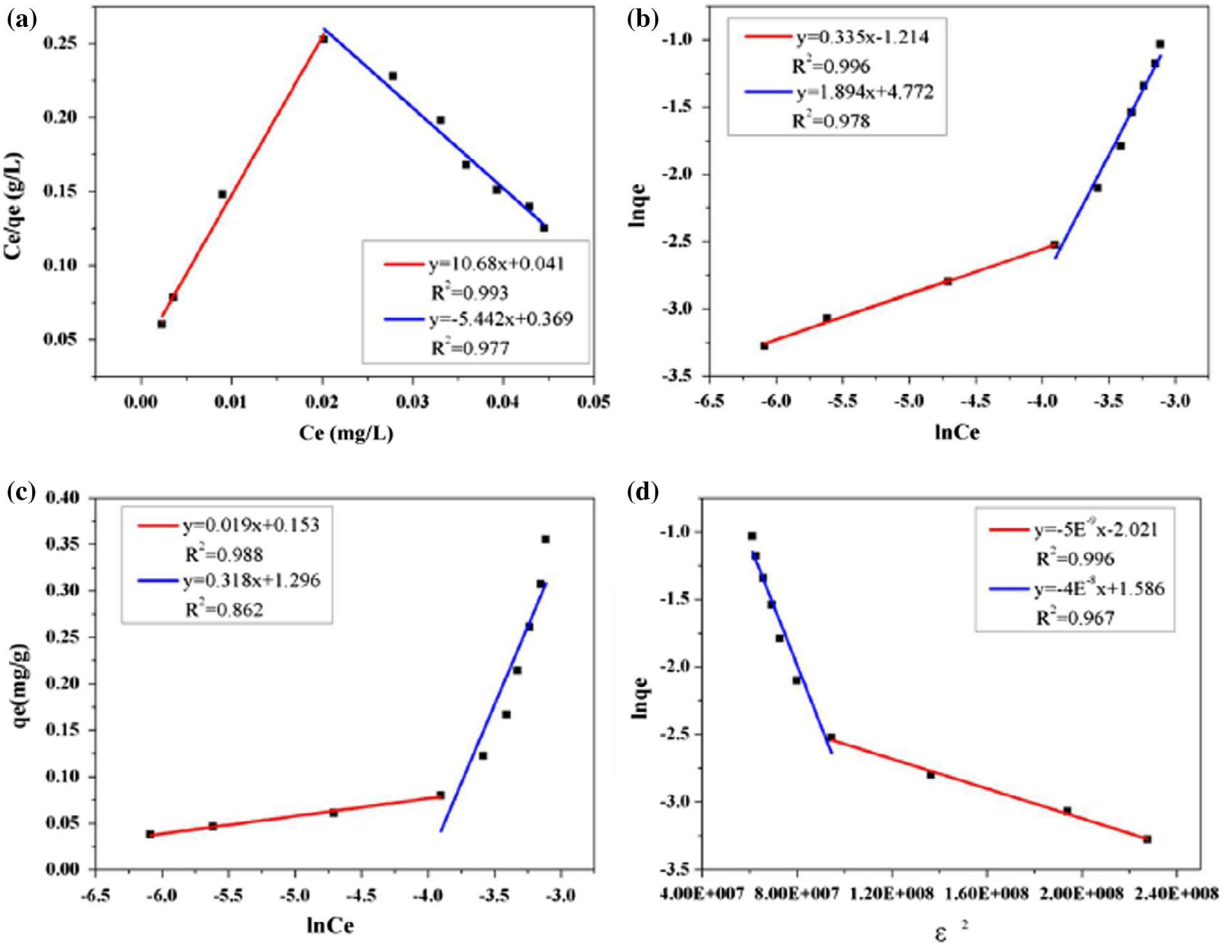
Adsorption isotherms of ANT fitting by two stages at 298 K, (a) Langmuir, (b) Freundlich, (c) Temkin, (d) D–R model.

In contrast to PHE, ANT showed an unusual phenomenon whereby two separate stages emerged for all the models fitted. Figure 7 shows an example at 298 K. This result suggests that the adsorption of ANT was complex and exhibited a “turning point” at a certain C_0_. This phenomenon was similar to Wang’s observations when the adsorbates’ concentrations were set high enough above their solubility levels.[10] The “turning point” in our research was estimated to correspond to solubility values of 0.7 mg l^−1^ for 298 and 303 K and 1.0 mg l^−1^ for 308 K. From the correlation coefficients derived for the four models, the Freundlich equation fit each stage of the isotherm well overall, and the regression parameters are presented in Table 4.

### 3.5. Thermodynamics studies

Adsorption thermodynamic studies were conducted at three temperatures of 298, 303, and 308 K for further analyzing the adsorption mechanism. From the results listed in Table 5, we can see the PHE and ANT results showed different trends. For PHE, the change in enthalpy (ΔH^0^) and entropy (ΔS^0^) are calculated as –51.29 kJ mol^−1^ and –0.18 kJ mol^–1^ K^−1^, respectively. From the negative ΔH, we can deduce that this is an exothermal process. The negative ΔS^0^ indicates a decreased randomness at the solid–liquid interface during the adsorption process, as the target molecules are fixed to the two-dimensional surface from the solution. The negative value of the Gibbs free energy (ΔG^0^) implies a spontaneous nature of PHE and ANT adsorption as well. The degree of spontaneity of the reactions decreased with increasing temperature, which is in agreement with the negative value of ΔH^0^ indicating this reaction is an exothermic process. Furthermore, the negative values of ΔG^0^, ranging from –0.84 to –2.48 kJ mol^−1^, indicated a feasible and spontaneous physical adsorption of PHE onto Fe@SiO_2_@PDA.

**Table 5.** Thermodynamic parameters of PHE and ANT.

Thermodynamic parameters	PHE		ANT	
		Whole	Low*	High*
ΔG^0^ (kJ mol^−1^)				
298 K	−2.40	−5.15	−11.4	−2.91
303 K	−1.55	−4.94	−10.1	−2.88
308 K	−0.735	−4.74	−8.96	−2.86
ΔH^0^ (kJ mol^−1^)	−51.2	−17.1	−81.5	−4.22
ΔS^0^ (kJ mol^−1^ K^−1^)	−0.164	−0.04	−0.235	−0.004

^*^ Low: C_0_ (initial concentration) < 0.1 mg l^–1^; high: C_0_ (initial concentration) ≥ 0.1 mg l^–1^.

As for ANT, two models were developed with consideration of two stages as was done for the isotherms calculation. This turning point was set at the solubility value just as had been done for the isotherm study. From the values presented in Table 3, we can deduce that this process is a spontaneous physical adsorption similar to the adsorption of PHE. The trend of ΔG^0^ at different temperatures is consistent with a positive value of ΔH^0^, indicating an exothermic adsorption, in accordance with Torabian’s research.[4] The negative ΔS^0^ reflects a property of decreased randomness.

### 3.6. Adsorption mechanism

In general, hydrophobic effects, electrostatic interactions, hydrogen bonding and π–π interactions are the most representative interaction mechanisms for adsorption of adsorbents in aqueous phase. With regard to the forces responsible for PAH adsorption, researchers have proposed many hypotheses and contributed to a large body of evidence. Yuan et al. [7] studied the interaction between the PAHs and porous carbon in water, and emphasized the role of π–π interactions,^[7]^ as was also postulated by Torabian et al. [4]. Araújo’s research [3] demonstrated that electrostatic interactions between the π-electrons of aromatic rings and adsorbents play a pivotal role^[3]^. To explain differences in adsorption between PHE and ANT, the π-electron number is not an influential factor, because both PAHs possess 14 π-electrons. From the information known about these two adsorbates, the greatest difference between PHE and ANT is in their solubility properties. Therefore, the hydrophobic effect should not be ignored. On the other hand, the adsorbates and our adsorbents both contain benzene rings leading use to deduce that π–π interactions between our adsorbents and adsorbate molecules should be relevant adsorption forces. In consideration of the properties of PHE and ANT and the thermodynamics parameters calculated, a physical adsorption process is implicated in which hydrogen bonding interactions are not involved. Taken together, our results indicate that the affinity of these adsorbents towards PAHs may not be straightforward, but may result from a combination of hydrophobic effects, π–π interactions, and van der Waals forces.[3,59]

### 3.7. Reusability

**Figure 8.**
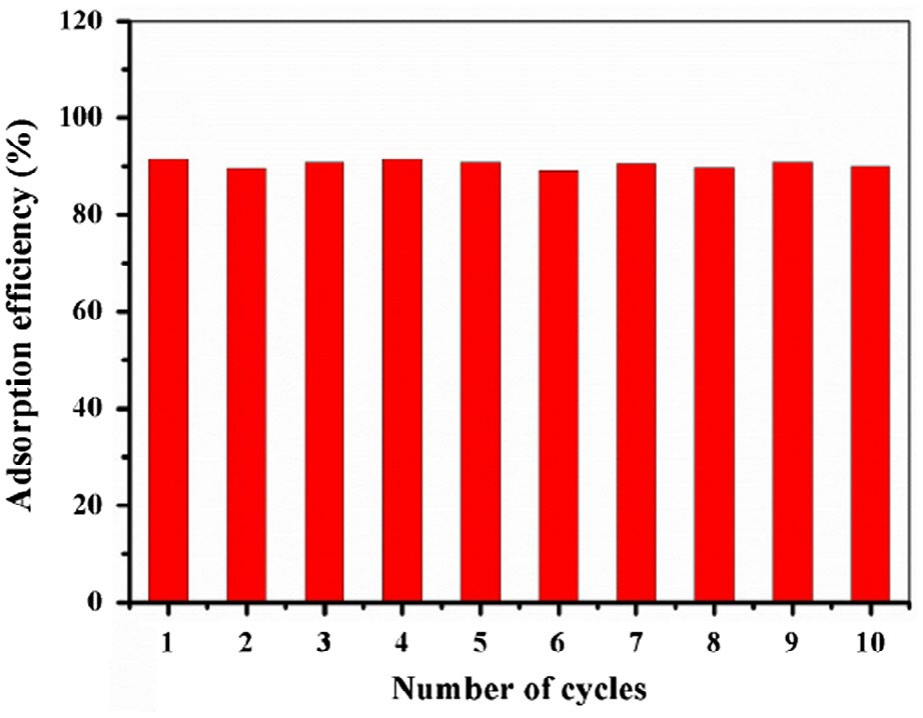
Recyclable adsorption of ANT by Fe@SiO_2_@PDA.

In order to investigate the regeneration of Fe@SiO_2_@PDA particles, they were washed with 1 ml methanol twice before reuse in the same adsorption system (298 K, 200 rpm for 1 h). As shown in Figure 8, these adsorbents can be recycled at least 10 times without obvious reduction in adsorption capacities. This property is a vital factor to reduce the overall cost of the application in pollutants removal. Up to now, some adsorbents such as carbon nanotube, graphene and so on have been utilized to remove pollutants with high efficiency, some of them provided better performance than Fe@SiO_2_@PDA, and others gave poorer removal rate than Fe@SiO_2_@PDA. However, Fe@SiO_2_@PDA material in this study exhibits some advantages such as easy separation, good reusability, low cost, simple operation and biocompatibility for environmentally friendly application. In general, we should make more effort to improve their merits and overcome their disadvantages to make further exploration, and develop more enhanced materials with larger specific surface area for such targets.

## 4. Conclusions

In summary, a magnetic Fe@SiO_2_@PDA composite was successfully fabricated using a simple low-cost two-step method and characterized by several techniques. The effective adsorption of PAHs to these particles in aqueous solution indicates this system should serve as a useful removal methodology for PAH pollutants. In order to elucidate the mechanism of adsorption, we compared the different adsorbance efficiencies of Fe@SiO_2_@PDA towards PHE and ANT. The results indicated that the adsorption process obeys pseudo-second-order kinetics and its isotherm fits best to the Freundlich model. The adsorption capacities for PHE and ANT were measured to be 0.185 mg g^−1^ and 0.367 mg g^−1^, respectively. Moreover, these processes are assumed to be exothermic, spontaneous physical adsorptions and are speculated to be dependent on hydrophobic effects, π–π interactions, and van der Waals forces.

Notably, even after 10 cycles, the adsorption efficiency barely decreased, indicating that Fe@SiO_2_@PDA would serve as a good reusable adsorbent. This is a valuable result which supports our rationale for making an iron-based magnetic polydopamine material to serve as a recyclable adsorbent for removal of PAHs from water. However, exploration of enhanced materials with higher specific surface area and higher selectivity are our future goals.

## Disclosure statement

No potential conflict of interest was reported by the authors.

## Funding

This work was supported by the National Natural Science Foundation of China [21377167].
